# Efficient Method for Photovoltaic Power Generation Forecasting Based on State Space Modeling and BiTCN

**DOI:** 10.3390/s24206590

**Published:** 2024-10-13

**Authors:** Guowei Dai, Shuai Luo, Hu Chen, Yulong Ji

**Affiliations:** 1 College of Computer Science, Sichuan University, Chengdu 610065, China; daigw@stu.scu.edu.cn (G.D.);; 2 National Key Laboratory of Fundamental Science on Synthetic Vision, Sichuan University, Chengdu 610065, China

**Keywords:** photovoltaic power forecasting, time series prediction, deep learning, intelligent fusion, state space model

## Abstract

As global carbon reduction initiatives progress and the new energy sector rapidly develops, photovoltaic (PV) power generation is playing an increasingly significant role in renewable energy. Accurate PV output forecasting, influenced by meteorological factors, is essential for efficient energy management. This paper presents an optimal hybrid forecasting strategy, integrating bidirectional temporal convolutional networks (BiTCN), dynamic convolution (DC), bidirectional long short-term memory networks (BiLSTM), and a novel mixed-state space model (Mixed-SSM). The mixed-SSM combines the state space model (SSM), multilayer perceptron (MLP), and multi-head self-attention mechanism (MHSA) to capture complementary temporal, nonlinear, and long-term features. Pearson and Spearman correlation analyses are used to select features strongly correlated with PV output, improving the prediction correlation coefficient (*R*^2^) by at least 0.87%. The K-Means++ algorithm further enhances input data features, achieving a maximum *R*^2^ of 86.9% and a positive *R*^2^ gain of 6.62%. Compared with BiTCN variants such as BiTCN-BiGRU, BiTCN-transformer, and BiTCN-LSTM, the proposed method delivers a mean absolute error (MAE) of 1.1%, root mean squared error (RMSE) of 1.2%, and an *R*^2^ of 89.1%. These results demonstrate the model’s effectiveness in forecasting PV power and supporting low-carbon, safe grid operation.

## 1. Introduction

Photovoltaic (PV) power generation technology is now widely used worldwide. The advancement of PV power generation technology has been a key driving force in clean energy. Technological progress has significantly enhanced the efficiency and cost-effectiveness of PV systems, offering strong support for the future of global sustainable energy [1]. However, the rapid growth of PV installations has come with challenges, particularly in power grid integration. The inherently unstable nature of PV output complicates effective dispatching, which poses new risks to grid stability [2,3]. Currently, global PV’s installed capacity is approaching 965 GW_p_, exacerbating these grid stability concerns [4]. To maintain grid stability, reliability, and efficiency, it is essential to exercise effective control over PV power generation [5]. In such cases, short-term forecasting has become increasingly important in PV power generation forecasting. Accurate PV generation forecasts not only optimize the operation of solar power systems but also enhance the reliability of the overall power grid [6]. For power companies that are reliant on PV energy, precise short- and long-term generation capability predictions are crucial. As a result, accurate forecasting of PV output has emerged as a primary research focus. PV energy forecasting is typically achieved through two main approaches: direct and indirect methods. The direct method forecasts PV power generation by directly reflecting the system’s generating capacity, as described in [7]. On the other hand, the indirect method predicts solar irradiance using meteorological data or weather forecasts and then inputs this information into PV system models to estimate power generation in the future. In some cases, such as when new PV systems lack historical data or when weather forecasts are needed for other purposes, the indirect method is often the more suitable strategy [8]. As these methods are effectively applied, PV forecast technology will continue to advance, ensuring stable operation and expanded PV system usage.

With the advances in deep learning, many deep learning-based models have been made in the field of renewable energy generation forecasting [9,10]. As an extension of artificial neural networks (ANNs), deep learning constructs multilayer neural networks to simulate the learning processes of the human brain, offering exceptional nonlinear modeling capabilities and adaptability. This provides deep learning with a marked advantage over traditional machine learning methods when processing complex time series data [11,12]. In PV power forecasting, especially within the indirect forecasting method, meteorological parameters are typically input as time series data, which imposes high demands on predictive models. Deep learning techniques can automatically extract valuable features and patterns from historical data, enabling the effective prediction of future energy demand or renewable energy generation [13,14,15]. This capability makes deep learning an ideal tool for forecasting PV power generation in highly dynamic and complex environmental conditions. Additionally, the application of deep learning is not confined to PV power forecasting but extends across other renewable energy sectors, including hydropower, biomass energy, wind energy, and solar thermal energy [16,17]. In these areas, deep learning methods can achieve high-accuracy power generation forecasts over particular time periods, significantly improving energy management efficiency and grid scheduling. The contributions of this paper are summarized as follows:

(1) BiTCN-MixedSSM model based on the Mamba state space model (SSM) is developed for PV power generation forecasting.
(2) The primary features and their interrelationships in PV generation data are analyzed using Pearson correlation and Spearman’s rank correlation coefficients, which enhances the accuracy of PV power forecasts.
(3) In order to address the problem of the dispersion of critical regional features, the correlated features identified through Spearman correlation coefficient analysis are integrated by the K-Means++ algorithm.
(4) Mixed-SSM core module comprising SSM, a multilayer perceptron (MLP), and a multi-head self-attention mechanism (MHSA) is proposed to enable the parallel fusion of multiple features.

The paper is structured as follows: Section 2 reviews relevant studies on machine learning and deep learning methods in PV power forecasting. Section 3 introduces the BiTCN-MixedSSM model, with a detailed explanation of the core Mixed-SSM architecture and the feature correlation analysis algorithm. Section 4 discusses the PV dataset, data preprocessing, and experimental setup. It also compares the performance of models that utilize Spearman’s coefficient for feature selection, highlighting the performance improvements gained through feature selection. A comprehensive qualitative comparison between the proposed method and the latest approaches is provided, followed by an analysis of the K-Means++ algorithm’s effectiveness in fusing different feature types. Finally, Section 5 presents the conclusions of this study.

## 2. Related Work

The application of machine learning models has become prevalent in the research of photovoltaic (PV) power generation. In the study by Bouchaib [18], both support vector machines (SVM) and Gaussian process regression (GPR) were employed to investigate the correlations between different input variables and solar PV energy output. The findings revealed that input data selection was more important than model choice. Markovics et al. [19] conducted a comparison of 24 straightforward machine learning (ML) models for day-ahead photovoltaic (PV) power prediction using numerical weather prediction (NWP). Their results highlighted that the selection of input data had a greater impact than the choice of model itself. Additionally, hyperparameter optimization was crucial for maximizing the potential of ML models, as the best RMSE without tuning was 3.1% higher than the optimized version. Robust models, such as MLP and gradient boosting regressor, were highly accurate even without tuning, whereas models like Lasso and decision trees required training to mitigate large errors due to default parameters. In the domain of PV power forecasting using deep learning models, Xiang et al. [20] introduced a hybrid approach that integrated temporal convolutional networks (TCN), gated recurrent units (GRU), and efficient channel attention networks (ECANet), which proved to be highly effective in improving the accuracy of short-term PV power predictions. Ma et al. [21] proposed a hybrid LSTM-TCN forecasting model, which also incorporated an NWP error correction mechanism and TimeGAN. This model’s performance was evaluated using data from a Chinese wind farm, successfully demonstrating the approach’s effectiveness.

Mo et al. [22] applied TCN and Prophet to forecast power load data in a separate study. The two models were combined using the least squares method to enhance prediction accuracy. Experimental results showed that the TCN-Prophet model outperformed conventional models, including ARIMA, RNN, LSTM, GRU, and various ensemble approaches, offering more reliable decision-making support for grid operations. Gao et al. [23] introduced a short-term power load forecasting technique utilizing a TCN-BiGRU-attention hybrid model, which was optimized using the Whale optimization algorithm (WOA). The findings indicated that this method delivered more precise short-term PV power forecasts. In another study, Mellit et al. [24] compared the performance of long short-term memory (LSTM), bidirectional LSTM (BiLSTM), gated recurrent unit (GRU), bidirectional GRU (BiGRU), and 1D convolutional neural networks for short-term PV power prediction. The models were evaluated across four time intervals—1 min, 5 min, 30 min, and 60 min—with *R*^2^ values ranging between 96.9% and 98%.

Particle swarm optimization (PSO) was applied by Zhang et al. [25] to find the optimal parameter combinations for variational mode decomposition (VMD), allowing automatic adjustments of energy data parameters across different features and reducing human error. A convolutional neural network (CNN) architecture was developed to balance operational efficiency and forecasting accuracy. The wild horse optimization (WHO) algorithm was then used to optimize the CNN’s hyperparameters, enhancing the model’s stability and accuracy, achieving a 72% and 79% improvement over a single CNN model. Accurate forecasting of wind and PV power generation enables timely scheduling and control of exchange power, preventing off-grid events caused by increased penetration of wind and solar energy, thus ensuring stable power system operation. Mohamed et al. [26] introduced a novel deep learning architecture, PV-Net, for short-term PV energy forecasting. The GRU unit gates were redesigned using dilated convolution operations to process historical PV time series, capturing spatial information while simulating temporal input sequence features. The GRU units were stacked bidirectionally to process PV data both forward and backward. The model achieved optimal performance with 5 residual conv-GRU modules per layer (MAE: 39.49%, RMSE: 81.03%).

Deep learning has emerged as the leading method for forecasting PV power generation. With the swift progress in deep learning technologies, numerous specialized submodules have been developed, and there is a growing trend toward incorporating these submodules into hybrid models. Zhang et al. [27] proposed an adaptive hybrid model where the original PV output was decomposed into regular and irregular components using IVMD. The regular components were forecasted with ARIMA, while the irregular components were predicted using IDBN.

Finally, the prediction results of each component were added to complete the final prediction results. This approach significantly enhanced short-term PV power forecasting, improving accuracy by 9–96% compared to other models. Mario et al. [28] proposed a CNN-LSTM hybrid model comprising five CNN and LSTM layers along with a fully connected layer, achieving RMSE and MAE values of 0.2923 and 0.0519, respectively. The K-Means++ algorithm was employed by Bai et al. [29] to classify weather types and generate distinct feature vectors by calculating statistical indicators from historical data. The Euclidean distance was then used to identify the weather type for the forecast day, and Grey relational analysis (GRA) and cosine similarity methods were applied to locate the most similar days. The LSTM neural network was subsequently used for power prediction, reducing RMSE by 66.73%, 70.22%, 65.59%, 70.51%, and 18.40% compared to other models, thus demonstrating superior predictive performance. Tang et al. [30] developed a hybrid PV power prediction model called ADBN, which integrated transfer learning. The model employed a parallel architecture combining a dilated convolutional neural network (DCNN) and a BiLSTM network to enhance feature representation. This parallel structure independently extracted spatial and temporal features from the input data. The results indicated that the transfer learning approach significantly improved both accuracy and training efficiency, reaching 69.51% and 71.42%, respectively. Wang et al. [31] introduced a GBDT-BiLSTM PV forecasting model that integrated a teacher-forcing mechanism with BiLSTM and an optimized GBDT model. This mechanism used actual power values to effectively calibrate the BiLSTM model, resulting in the lowest MAE (0.08), MSE (0.151), and MASE (0.05) values, showcasing excellent prediction accuracy. Geng et al. [32] applied the Time2Vec technique to break down time series data into periodic and non-periodic components for more efficient feature extraction. A hybrid model incorporating a wide first-layer kernel deep CNN (WDCNN) and BiLSTM was proposed, where WDCNN leveraged a large receptive field to extract valuable information, and stacked BiLSTM layers captured temporal dependencies from both past and future datasets. This approach led to improved wind power forecasting accuracy by 30.59%, 42.78%, 24.35%, and 30.59%, respectively. Akhter et al. [33] utilized the Salp swarm algorithm (SSA) to optimize the hyperparameters of an RNN-LSTM model. The SSA-RNN-LSTM model outperformed other methods, achieving the lowest RMSE and MSE values, the highest *R*^2^, and the fastest convergence rate.

## 3. The Proposed Work

### 3.1. BiTCN-MixedSSM

**Figure 1:**
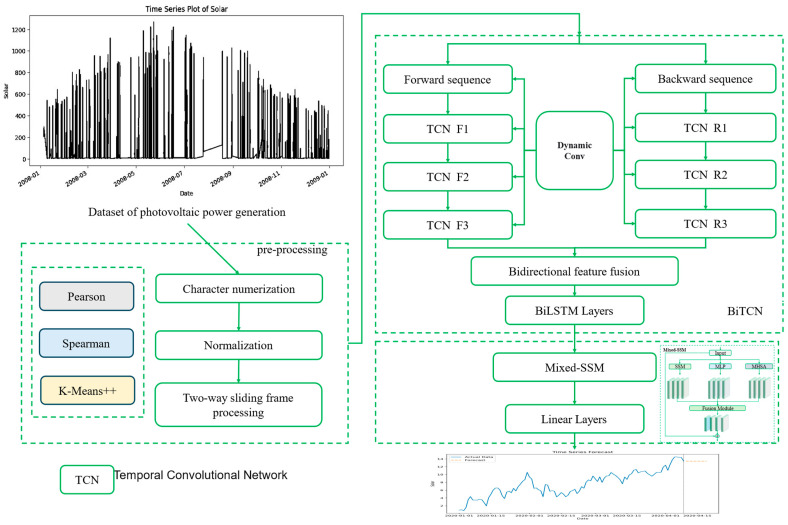
Detailed diagram of the proposed BiTCN-MixedSSM model structure.

The BiTCN-MixedSSM model is composed of a bidirectional temporal convolutional network (BiTCN), dynamic convolution (DC) [34], BiLSTM, and the proposed mixed state space model (Mixed-SSM). The structure of the model is illustrated in Figure 1. The components and workflow are as follows. Data input and preprocessing: The model uses time series data from PV power generation as input. The top-left corner of the figure displays a sample dataset showing the variation in solar power generation over time. The data undergoes a preprocessing phase, which includes character numericalization, converting non-numeric data into numeric form. Normalization: Standardizing the data range to stabilize model training. Bidirectional sliding window processing; this involves creating fixed-size input sequences while considering both past and future time steps. To optimize important features, the preprocessing stage also applies the K-Means++ algorithm for feature fusion [35]. Feature selection is conducted using Pearson correlation coefficients and Spearman coefficients before the fusion [36]. The preprocessed data is then passed into the BiTCN module, which serves as the core component of the model. It consists of two main branches; Forward sequence: This branch contains three stacked TCN layers (F1, F2, and F3). Backward sequence: Similarly, the backward sequence contains three TCN layers (R1, R2, and R3), but processes the reverse time sequence. Dynamic convolution is employed to improve the convolution operations used in both the forward and backward sequences of the BiTCN-MixedSSM model. This allows the kernel size or shape to change dynamically across different input time series data, accommodating sequences of varying lengths and structures. The two branches are connected by a dynamic convolution layer, enabling information flow between the forward and backward sequences. The BiTCN architecture is designed to capture temporal dependencies in both forward and backward directions within the data, allowing for a more comprehensive analysis of sequential patterns. The BiTCN is integrated with bidirectional feature fusion and BiLSTM. The output of BiTCN passes through a bidirectional feature fusion layer, where features from the forward and backward sequences are combined into a unified representation. The BiLSTM is mainly employed to improve the extraction of periodic features from time series data, utilizing multiple layers akin to the BiTCN structure to capture contextual information at any point in the sequence. This combined information is then passed into the Mixed-SSM, which is designed to capture complex interactions between various positions within the sequence. The Mixed-SSM interprets the significance of each element in the sequence. After undergoing linear processing, the model makes its final predictions.

### 3.2. Mixed-SSM Module

**Figure 2:**
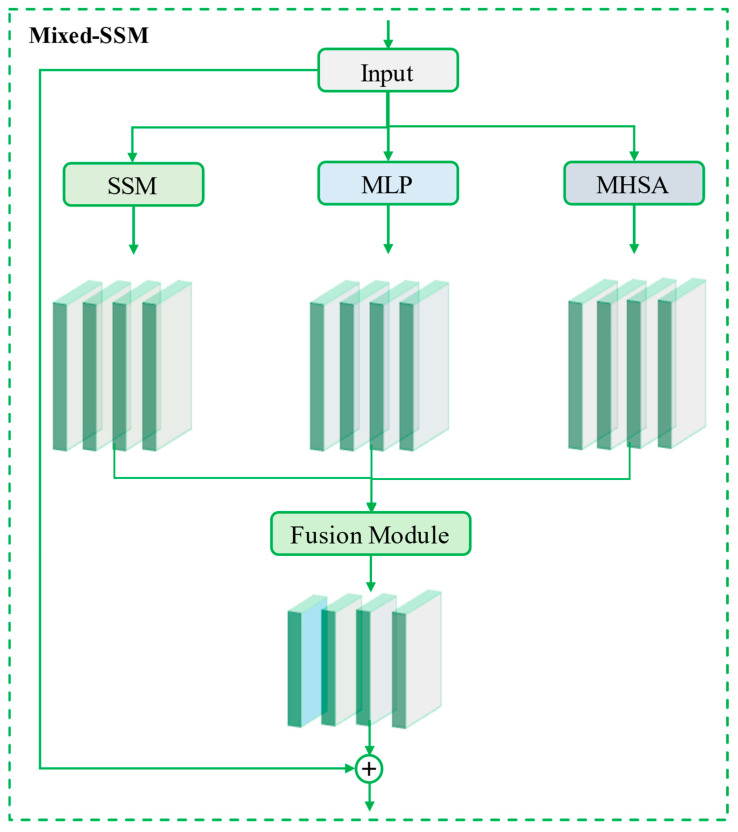
Diagrammatic representation of the structure of the proposed novel Mixed-SSM module.

The core module of the BiTCN-MixedSSM model, Mixed-SSM, introduces an innovative enhancement to the feature extraction structure, BiTCN, by enabling parallel fusion of multiple features. The core architecture of the model is composed of the state space model (SSM) module, the multilayer perceptron (MLP) module, and the multi-head self-attention (MHSA) module, as shown in Figure 2. The features extracted by these three parallel modules are fused through accumulation, followed by skip connections, similar to the ResNet architecture, to reconnect and optimize the fused features. The mathematical equation governing the entire module is expressed in Equation (1), where X represents the input, Y the output, *F* the transformation function, and *θ* the set of all learnable parameters. *F* can be further expanded, as shown in Equation (2), and the final representation of the entire Mixed-SSM is given in Equation (3).

(1) Y=F(X;θ)+X

(2) $$F\left(X;\theta \right)=Fusion\left(f_{SSM}\left(X;\theta_{SSM}\right),f_{MLP}\left(X;\theta_{MLP}\right),f_{MHSA}\left(X;\theta_{MHSA}\right);\theta_{Fusion}\right)$$

(3) $$Y=Fusion(f_{SSM}(X;\theta_{SSM}),f_{MLP}(X;\theta_{MLP}),f_{MHSA}(X;\theta_{MHSA});\theta_{Fusion})+X$$

The core architecture of the Mixed-SSM is designed based on the Mamba neural network model. The Mamba model leverages the theoretical framework of the state space model (SSM) and utilizes selective state spaces to enhance the efficiency and effectiveness of capturing long-sequence information [37]. This design overcomes the limitation of transformers, which are unable to achieve linear time complexity when processing long sequences, enabling the model to process entire sequences in parallel. As a result, the overall processing capacity is significantly improved.

**Figure 3:**
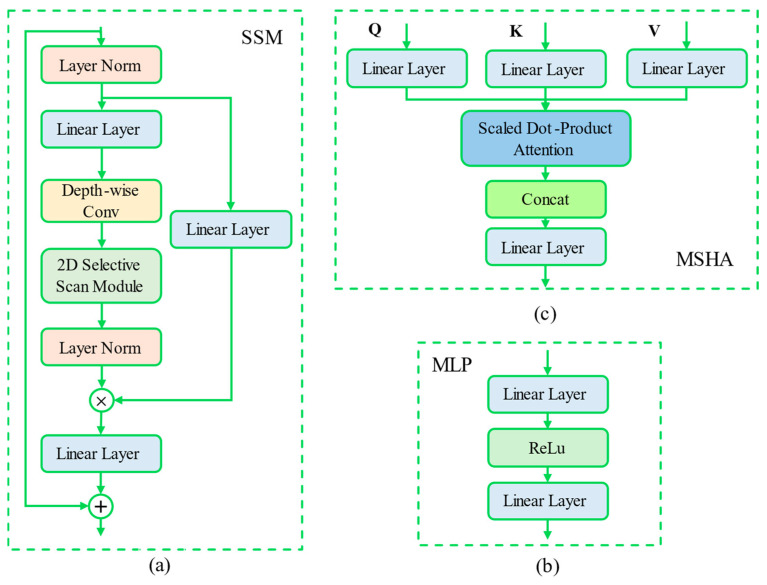
Diagrammatic representation of the detailed structure of the proposed Mixed-SSM module with (**a**) state space model, (**b**) multilayer perceptron, and (**c**) multi-attention mechanism.

The state space model (SSM) introduces a unique cross-scanning module (CSM) designed to enhance direction-sensing capabilities and improve computational efficiency. As illustrated in Figure 3a, SSM plays a critical role in modeling dynamic visual systems by capturing changes in time series and using observation-generated equations for modeling. The mathematical expression of the observation equation is shown in Equation (4), where $x_{t}$ represents the system state at time t, $u_{t}$ is the control signal, and $w_{t}$ denotes noise in the state transition process, reflecting uncertainties in state changes. Simultaneously, the observation equation is defined by Equation (5), where $y_{t}$ is the observation at time t, and $v_{t}$ represents observation noise, indicating the discrepancy between the model prediction and the actual observation. Matrices A, B, C, and D collectively define the system dynamics, organically linking state transitions with observations. Additionally, the CSM effectively addresses directional sensitivity by reorganizing visual features into ordered block sequences, as shown in Equation (6). This process involves specific handling of the input visual features *v_t_*, enabling the model to more effectively manage spatial information in dynamic scenes, thereby enhancing its adaptability and processing capability.

(4) $$x_{t+1}=A\cdot x_{t}+B\cdot u_{t}+w_{t},$$

(5) $$y_{t}=C\cdot x_{t}+D\cdot u_{t}+v_{t},$$

(6) $$CSM\left(V\right)=Order\left(Traverse\left(V\right)\right).$$

The multilayer perceptron (MLP) consists of NNN linear layers, each equipped with learnable weights, bias parameters, and activation functions, as shown in Figure 3b. In recent years, the widespread application of the vision transformer (ViT) has demonstrated a significant impact; however, the MLP-Mixer offers a novel perspective by replacing the transformer in ViT with the MLP module of the mixer model [38]. This substitution reduces the degrees of freedom during the feature extraction process and introduces an innovative approach by alternating the exchange of information between and within patches. This alternating mechanism significantly improves model performance, further validating the potential and value of pure MLP architectures in handling complex visual tasks. Additionally, the structural design of the MLP-Mixer reveals that, even without self-attention mechanisms, the MLP architecture can efficiently process large-scale visual data, offering new directions for future model designs.

In the self-attention mechanism, multi-head self-attention (MHSA) introduces several attention heads, enabling each head to independently learn and represent unique attention patterns within different subspaces. These distinct representations are then combined to provide a more comprehensive understanding of the relationships within the input data. This design enables MHSA to capture data information more flexibly, particularly by focusing on and processing various parts of sequential data with greater precision [39]. As illustrated in Figure 3c, MHSA transforms the input tensor X into three matrices: query (Q), key (K), and value (V), through linear projections. These matrices are then split into n sets of distinct $Q_{i}$, $K_{i}$ and $V_{i}$, where each set represents an independent attention head $Head_{i}$. Each head performs self-attention calculations within its own subspace, generating n different output matrices. These output matrices are then concatenated to form a unified attention representation. The self-attention calculation is described by Equation (7), with the computation for each attention head given by Equation (8), and the combination of heads by Equation (9). The MHSA design enhances the model’s ability to capture complex data patterns while providing greater flexibility, allowing for a more detailed analysis of the internal relationships within sequential data. This extends the potential applications of the self-attention mechanism.

(7) $$Attention(Q,K,V)=softmax(\frac{QK^{T}}{\sqrt{d_{k}}})V$$

(8) $$Head_{i}=Attention(Q_{i},K_{i},V_{i})$$

(9) $$Out=concat(Head_{1},Head_{2},\cdots ,Head_{n})$$

### 3.3. Correlation Analysis

#### 3.3.1. Pearson Coefficient

PV power forecasting utilizes various input features, and it is essential to assess the correlation of these features with PV power generation to confirm their effectiveness. The Pearson correlation coefficient (PCC) is a widely adopted method for determining the correlation between multiple variables. By calculating the PCC between the chosen features and the actual PV power output, the relevance and effectiveness of the features can be evaluated. The formula for the Pearson correlation coefficient is as follows:

(10) $$P_{xy}=\frac{\sum_{i=1}^{n}\;(x_{i}-\bar{x})(y_{i}-\bar{y})}{\sqrt{\sum_{i=1}^{n}\;{\left(x_{i}-\bar{x}\right)}^{2}}\sqrt{\sum_{i=1}^{n}\;{\left(y_{i}-\bar{y}\right)}^{2}}}$$

The term $P_{xy}$ represents the correlation coefficient. When the absolute value of the coefficient approaches 1, it indicates a strong correlation between the two variables, with the sign denoting either a positive or negative relationship. In the formula, $x_{i}$ and $y_{i}$ represent the *i*-th observations of the two variables, while $\bar{x}$ and $\bar{y}$ denote their respective mean values.

**Figure 4:**
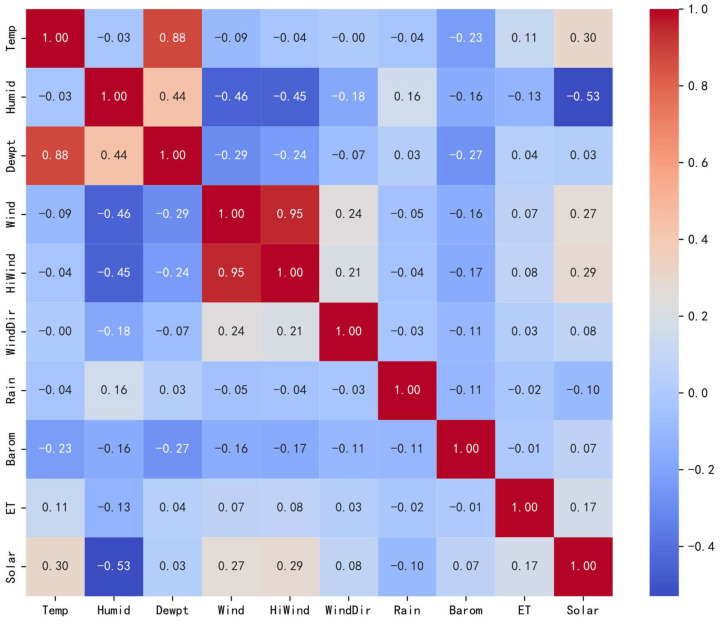
Graphical representation of Pearson correlation analysis of PV power forecast data.

The correlation coefficients for 10 input features are calculated using Equation (10), as shown in Figure 4. The findings suggest an inverse relationship between PV output and both humidity and precipitation. In contrast, a direct relationship is found with factors such as temperature, dew point, wind speed, maximum wind speed, wind direction, atmospheric pressure, and surface evapotranspiration. Among these, temperature, wind speed, maximum wind speed, and surface evapotranspiration strongly influence PV output, whereas dew point, wind direction, and pressure have a relatively smaller effect. In practice, high wind speeds aid in cooling PV panels, thereby improving solar energy utilization efficiency. Although high temperatures can enhance photoelectric conversion, the peak temperature typically occurs after the peak PV generation, indicating a nonlinear relationship between temperature and power generation. Considering these factors, temperature and wind speed significantly affect PV output. To improve the accuracy of the PV power prediction model, humidity and precipitation can be excluded, while the remaining seven features should be retained as input variables.

#### 3.3.2. Spearman Coefficient

The Spearman correlation, much like the Pearson correlation coefficient (PCC), evaluates the strength of the association between two variables; however, it operates on ranked data rather than raw values. To calculate the Spearman rank correlation coefficient for two random variables, X and Y, the following formula is used:

(11) $$Spe\left(X,Y\right)=1-\frac{6\sum_{i=1}^{N}\;{\eta_{i}}^{2}}{N\left(N^{2}-1\right)}.$$

If there is no association between X and Y, meaning |{x_1,…,x_*N*}| = *N* = |{y_1,…,y_*N*}|, the Spearman rank correlation coefficient is computed using Equation (11). However, if X and Y contain identical values, the Spearman coefficient defaults to the Pearson coefficient, as shown in Equation (12). *Rank*(X′) and *Rank*(Y′) must be adjusted using local corrections, with identical values assigned the arithmetic mean of their ranks. Thus, based on *Rank*(X′) and *Rank*(Y′), the Spearman correlation coefficient for X and Y is computed as the ratio between the covariance of the rank vectors and the product of their standard deviations.

(12) $$\begin{array}{c} Spe\left(X,Y\right)=Pearson\left(Rank\left(X^{\prime }\right)\right),\\ Rank\left(Y^{\prime }\right)=\frac{Cov(Rank(X^{\prime }),Rank(Y^{\prime }))}{S_{Rank(X^{\prime })}S_{Rank(Y^{\prime })}}, \end{array}$$

In the equation, when $\bar{Rank\left(X^{\prime }\right)}=\frac{1}{N}\sum_{i=1}^{N}\;$ and $\bar{Rank\left(Y^{\prime }\right)}=\frac{1}{N}\sum_{i=1}^{N}\;$, the values are induced by $t_{i}$.

The Spearman coefficient $Spe\left(X,Y\right)$ can take positive, negative, or zero values, indicating the nature of the relationship between X and Y. It can be determined using two methods, corresponding to Equations (11) and (12).

**Figure 5:**
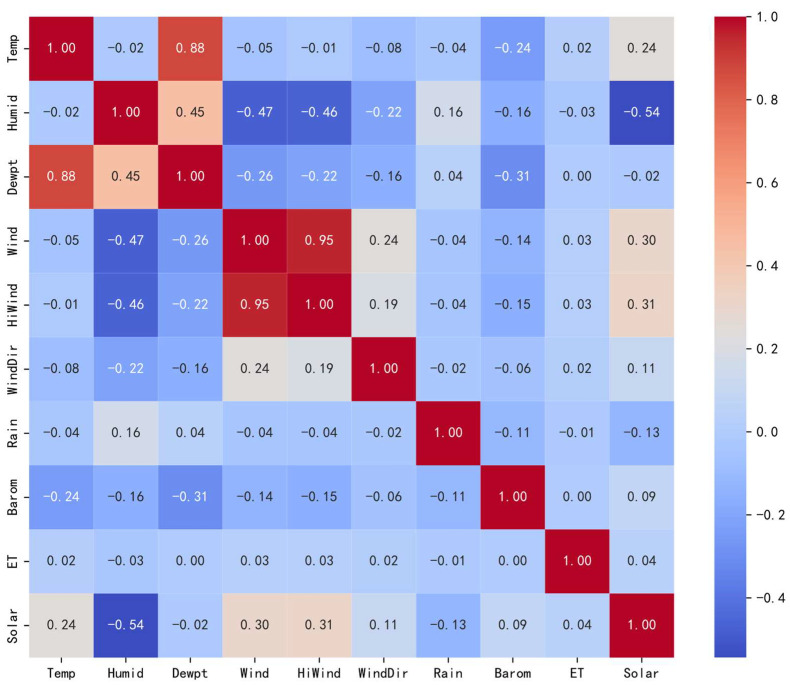
Graphical representation of Spearman’s coefficient correlation analysis of PV power forecast data.

The Spearman coefficient equation is applied to calculate the correlation coefficients of 10 input features, with the results shown in Figure 5. These results are consistent with those obtained using the Pearson correlation coefficient. A negative correlation is observed between PV output and both humidity and precipitation. As this correlation approaches −1, it indicates an increasingly stronger negative impact on PV generation. This underscores Spearman’s ability to handle monotonic trends in correlation analysis. When examining the variables, temperature exhibits a positive correlation with dew point temperature, surface evapotranspiration, and PV generation. Humidity is positively correlated with both precipitation and dew point temperature. Dew point temperature is positively associated with temperature, humidity, and precipitation but shows no correlation with surface evapotranspiration. Wind speed is positively correlated with maximum wind speed, wind direction, surface evapotranspiration, and PV output. Both maximum wind speed and wind direction are consistent with wind speed. Precipitation is positively correlated with humidity and dew point temperature. Atmospheric pressure positively correlates with PV output but has no relationship with surface evapotranspiration. Surface evapotranspiration is positively correlated with temperature, wind speed, maximum wind speed, wind direction, and PV output, while it shows no correlation with dew point temperature or atmospheric pressure.

## 4. Experimental Results and Discussion

### 4.1. Introducing the Datasets

**Figure 6:**
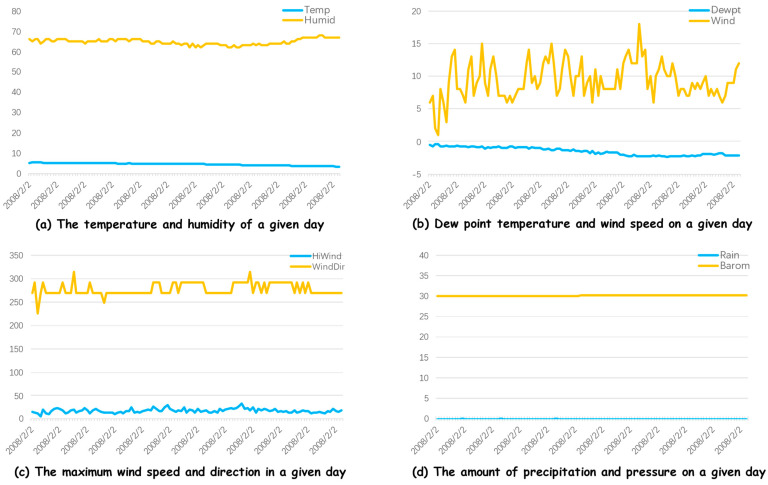
The dataset of PV power generation in relation to meteorological parameters presents various curves, with the y-axis indicating the range of values corresponding to each curve. These curves include: (**a**) the temperature versus humidity relationship, (**b**) the dew point temperature plotted against wind speed, (**c**) maximum wind speed in relation to wind direction, and (**d**) precipitation versus barometric pressure for a specific day analyzed in the study.

The dataset utilized in this research is obtained from the U.S. National Weather Service (NWS) and the University of Management and Technology (UMT). It includes PV power generation data along with relevant meteorological parameters collected in Amherst, Massachusetts, during the year 2008. The dataset encompasses 10 variables: timestamp, temperature (Temp), humidity (Humid), dew point temperature (Dewpt), wind speed (Wind), maximum wind speed (HiWind), wind direction (WindDir), precipitation (Rain), barometric pressure (Barom), surface evapotranspiration (ET), and PV generation (Solar). This study aims to forecast PV output by examining meteorological factors such as temperature, humidity, and dew point temperature. The dataset consists of 12,355 entries for the training set, 3524 for the test set, and 1739 for the validation set. The proposed model and related approaches are evaluated for their prediction accuracy in PV generation based on experimental results. Figure 6a–d displays the trends of two randomly selected meteorological parameters over a specific day. The data indicate relatively stable temperatures with a slight decline, while humidity fluctuates, dropping slightly around midday. Dew point temperature also fluctuates and shows a downward trend. Wind and maximum wind speeds vary significantly, indicating alternating strong and weak winds, while wind direction remains relatively constant. Precipitation is almost zero, and barometric pressure shows little fluctuation but trends upward overall. Figure 6a illustrates two lines: the blue line, which remains relatively stable, represents temperature, maintaining a constant value of around 20 units. In contrast, the yellow line represents humidity, fluctuating between 60 and 80 units over the course of the day. Figure 6b shows the blue line, representing dew point temperature, remaining stable at around 5 units. The yellow line, indicating wind speed, shows notable variability, with a peak near 20 units and a low close to 5 units. Figure 6c illustrates wind characteristics. The blue line, likely representing wind direction, remains constant around 100 units, while the yellow line, representing maximum wind speed, shows irregular fluctuations between 200 and 250 units. Figure 6d displays two nearly flat lines: the blue line, possibly indicating pressure, remains constant at around 5 units, and the yellow line, likely representing precipitation, remains stable at around 30 units throughout the observation period.

### 4.2. Dataset Preprocessing

The PV power generation prediction dataset, which integrates PV output with meteorological parameters, consists of historical sensor data recorded at timestamp intervals. The data collection starts on 5 January 2008, at 9:50 a.m., and ends on 31 December 2008, at 4:25 p.m., with readings taken at 5-min intervals. The time-series dataset includes input features, target predictions, and input sequence length. While the timestamp is not used as an input for prediction, it is crucial for subsequent processing and correlation analysis. To facilitate this, the timestamp is split into separate components—year, month, day, hour, minute, and second—and an additional column, Time Of Day, is added to record the hour and compute the total minutes. The split timestamp aligns with the original data columns. Data normalization is applied to convert entries from different ranges into a consistent scale to reduce regression errors while maintaining correlations between dataset features. Z-score normalization is employed to rescale the data, setting the mean to zero and the standard deviation to one. The formula for Z-score normalization is represented as follows:

(13) $$\mu =\frac{1}{n}\sum_{i=1}^{n}\;x_{i}$$

(14) $$\sigma =\sqrt{\frac{1}{n-1}\sum_{i=1}^{n}\;(x_{i}-\mu {)}^{2}}$$

(15) $$z_{i}=\frac{x_{i}-\mu }{\sigma }$$

**Figure 7:**
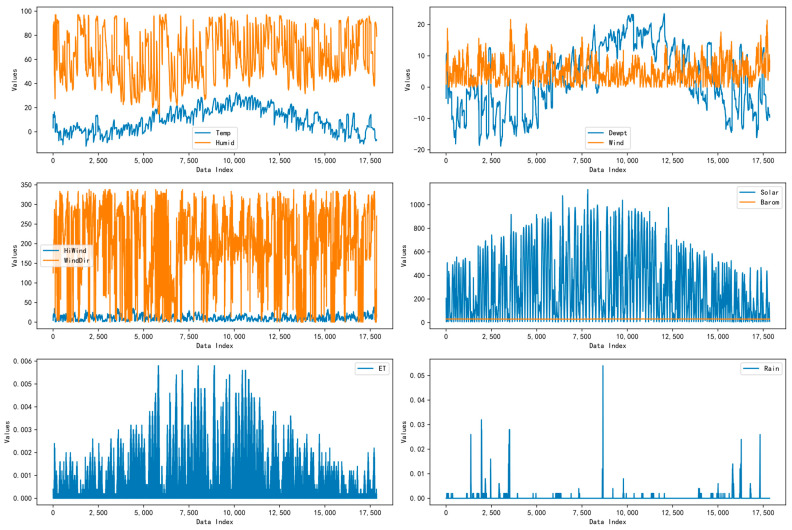
Graphical representation of the unstandardized PV forecast dataset.

**Figure 8:**
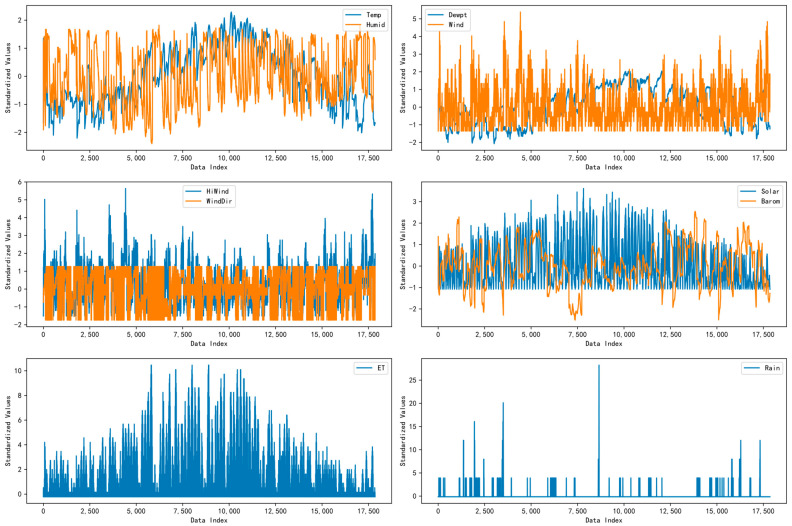
Graphical representation of a standardized PV power forecasting dataset.

In the equation, μ represents the mean of the original data, σ denotes the standard deviation of the original data, $z_{i}$ is the standardized value of the *i*-th observation $x_{i}$, and $x_{i}$ refers to the original feature’s *i*-th observation. Before feeding the data into the model, all feature values should be normalized. Figure 7 and Figure 8 illustrate the distributions of each feature’s original and normalized data.

### 4.3. Experimental Details

The BiTCN-MixedSSM model is implemented using the PyTorch 2.1.1 deep learning framework, with PyTorch running on CUDA version 11.8 and leveraging the cuDNN library for GPU acceleration. During the pre-training phase, a batch size of 64 is used, and the model is trained for 150,000 steps. The AdamW optimizer is employed with an initial learning rate set to 1.0 × 10^3^, using a linear learning rate adjustment strategy. For fine-tuning, the batch size is adjusted to 34, and the learning rate is set to 2.0 × 10^3^. All models are trained for 120 epochs on the training data, with checkpoints saved for the best accuracy on the validation set, followed by evaluation on the test set. The experiments are conducted on an Ubuntu 22.04 server equipped with an Intel Xeon Gold 6240 CPU @ 2.60 GHz and an NVIDIA GeForce GTX 3090Ti GPU (24 GB).

To evaluate the prediction performance, three widely used metrics are selected: root mean square error (RMSE), mean absolute error (MAE), and the coefficient of determination (*R*^2^). RMSE is more sensitive to outliers and reflects the standard deviation of prediction errors, while MAE measures the absolute error loss. *R*^2^ indicates the goodness of fit between the predicted and actual values. The model assessment metrics in this study are normalised to between 0 and 1. These metrics can be given by the following formula:

(16) $$MAE=\frac{1}{N}\sum_{i=1}^{N}\;|y(i)-y_{p}(i)|$$

(17) $$RMSE=\sqrt{\frac{1}{N}\sum_{i=1}^{N}\;{\left(y(i)-y_{p}(i)\right)}^{2}}$$

(18) $$R^{2}=1-\frac{\sum_{i=1}^{N}\;{\left(y(i)-y_{p}(i)\right)}^{2}}{\sum_{i=1}^{N}\;{\left(y(i)-\bar{y}\right)}^{2}}$$

(19) $$x_{\mathrm{normalized}}=\frac{x-x_{min}}{x_{max}-x_{min}}$$

In these equations, y(i) and $y_{p}(i)$ represent the true value and predicted value, respectively, at the $i^{th}$ timestamp. $\bar{y}$ denotes the mean of the actual power generation, and N is the number of prediction points. The RMSE provides an intuitive measure of error magnitude with a range of (0, +∞). When the true and predicted values are identical, RMSE equals zero, indicating a perfect model. Similarly, the MAE equals zero when the predicted and actual values are identical, also representing a perfect model. The *R*^2^ score (where R is the correlation coefficient and *R*^2^ is the coefficient of determination) indicates prediction accuracy; when *R*^2^ is close to 1, the model’s predictive accuracy is higher. Performance metrics normalized equation where x is the original value, $x_{min}$ is the minimum value in the dataset and $x_{max}$ is the maximum value in the dataset.

### 4.4. Spearman Feature-Based Correlation Analysis

In this section, the effectiveness of using the Spearman coefficient to filter input feature sets is validated, aiming to reduce the model’s input dimensions while preserving its generalization ability and accuracy. To evaluate this approach, comparisons are made across multiple models, including long short-term memory (LSTM), 1D convolutional neural network (1D CNN), backpropagation neural network (BPNN), decision tree (DT), random forest (RF), XGBoost, and LightGBM. All deep learning models are configured with the same optimizer, learning rate, and training epochs, whereas non-deep learning models are trained until they reach convergence. Each algorithm is trained and tested on identical datasets, and their performance is assessed using the same evaluation metrics to ensure a fair comparison. The selected input features include temperature, wind speed, maximum wind speed, wind direction, barometric pressure, evapotranspiration, and PV output, resulting in six input features and one target variable.

The test results on the dataset are shown in Table 1. Among the machine learning models, XGBoost achieved the best performance, with MAE and RMSE values of 0.347 and 0.269, respectively, and an *R*^2^ score of 0.689. It demonstrated higher prediction accuracy than RF and DT. LightGBM performed second best, with XGBoost improving *R*^2^ by 2.074% compared to LightGBM. In the neural network comparison, the proposed BiTCN-MixedSSM model outperformed others, with MAE and RMSE values of 0.011 and 0.012, respectively, and an *R*^2^ score of 0.891. LSTM was the second-best, with BiTCN-MixedSSM improving *R*^2^ by 9.73% compared to LSTM. BPNN showed the lowest performance, with an *R*^2^ score of 0.709. In summary, BiTCN-MixedSSM achieved the best performance for PV output prediction using the selected features. Notably, LSTM, despite its simpler structure, performed well, highlighting its advantage in handling time series data.

### 4.5. Spearman PV Power Prediction

The Spearman coefficient enhances the Pearson correlation coefficient by shifting the focus from linear relationships to monotonic relationships. While the Pearson coefficient quantifies the strength of a linear association between variables, the Spearman coefficient evaluates how well a monotonic function can describe the relationship between two variables, regardless of whether it is linear or not. Based on the conclusions drawn in Section 3.3.2 regarding the Spearman coefficient, several experiments were conducted for feature selection. In Experiment Group 1, the original features were used, including temperature, humidity, dew point, wind speed, maximum wind speed, wind direction, precipitation, barometric pressure, evapotranspiration, and PV output. In Group 2, the humidity was excluded, and Group 3 omitted the dew point. Group 4 excluded precipitation. In Group 5, both humidity and dew point were excluded, while Group 6 excluded humidity and precipitation. In Group 7, dew point and precipitation were excluded, and Group 8 excluded all three negatively correlated features: humidity, dew point, and precipitation.

The proposed BiTCN-MixedSSM model was used for all experiments, and the results are presented in Table 2. In Experiment Groups 2 to 4, selectively removing one of the negatively correlated features (humidity, dew point, or precipitation) resulted in improved performance. Group 3, which removed the dew point, showed the most significant improvement, with an increase in *R*^2^ of 1.37%. Groups 2 and 4 also improved by 1.249% and 0.87%, respectively. In Experiment Groups 5 to 7, removing two of the negatively correlated features further enhanced performance. Group 5 and Group 6 showed the most substantial improvements, consistent with the findings from Group 3, where dew point removal yielded better results. Group 5 achieved MAE and RMSE values of 0.045 and 0.065, respectively, with a 3.12% improvement in *R*^2^ compared to Group 1 and a 1.72% improvement compared to Group 3. Experiment Group 8, which removed all three negatively correlated features, achieved the best performance among all groups, with an MAE of 0.011, RMSE of 0.012, and an *R*^2^ score of 0.891. This represents an 11.23% improvement in *R*^2^ over Group 1, a 9.72% improvement over Group 3, and a 7.87% improvement over Group 5.

### 4.6. Generation Forecasting with Full Input Features

To assess the performance of the BiTCN-MixedSSM model and its related models for PV power prediction, experiments are conducted using a dataset that incorporates all input features. This comprehensive approach thoroughly evaluates the model’s predictive capabilities across various factors influencing PV output. The models tested include traditional methods such as RNN, LSTM, ARIMA, and GARCH, as well as hybrid models like CNN-LSTM, which serves as a representative of mixed models. The proposed BiTCN-MixedSSM builds upon the BiTCN framework, so additional models like BiGRU, SVM, Transformer, and LSTM are optimized for comprehensive comparison. The results are presented in Table 3. The data in Table 3 shows that the BiTCN-MixedSSM model outperforms the other nine models in terms of MAE, RMSE, and *R*^2^. This demonstrates that the BiTCN-MixedSSM model more effectively captures the complex relationships between PV power generation and meteorological parameters, outperforming the other models in this study. Among traditional methods, LSTM performs best, with an *R*^2^ of 0.705. The hybrid CNN-LSTM model, which combines CNN and LSTM, shows improved performance with an *R*^2^ increase of 0.85% over LSTM. Within the BiTCN variants, the BiTCN-Transformer model achieves the best performance, with an MAE of 0.098, RMSE of 0.147, and *R*^2^ of 79.2%. The BiTCN-Transformer outperforms other BiTCN variants. The BiTCN-MixedSSM model demonstrates a 21.43% reduction in RMSE and a 1.14% improvement in *R*^2^ compared to the BiTCN-Transformer. Among all BiTCN variants, the BiTCN-MixedSSM achieves the best overall performance, with an MAE of 0.077, RMSE of 0.112, and *R*^2^ of 80.1%. The prediction of photovoltaic power generation based on the corresponding relationships indicates that the BiTCN-MixedSSM offers superior accuracy compared to nine other models. Therefore, this model is recommended for PV power generation forecasting.

**Figure 9:**
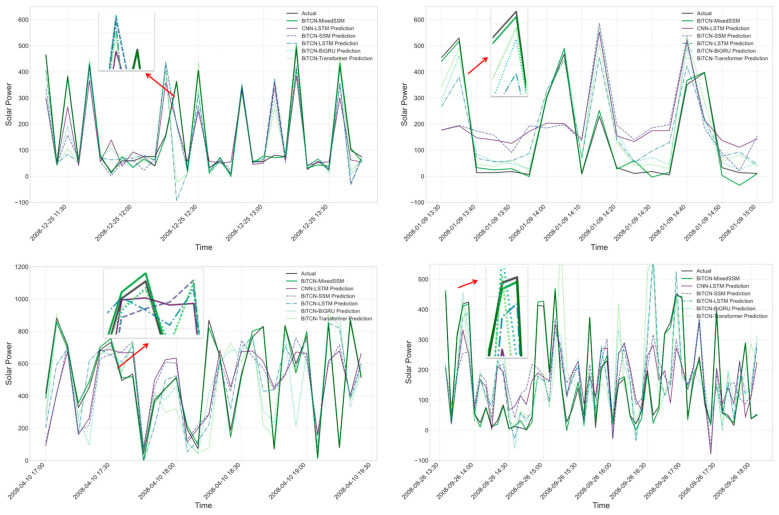
Comparative graphical representation of actual and predicted PV generation for BiTCN variants.

In the comparison between CNN-LSTM and standard BiTCN variants optimization, four random data intervals were selected for validation: 25 December 2008, from 11:30 to 13:30; 5 January 2008, from 09:50 to 9 January 2008, at 15:00; 10 April 2008, from 07:00 to 19:25; and 22 September 2008, from 07:05 to 26 September 2008, at 18:05. As shown in Figure 9, Our experimental results demonstrate the comparative performance of various models in predicting solar power generation, encompassing the actual values and predictions from BITCN-MixedSSM, CNN-LSTM, BITCN-SSM, BITCN-LSTM, BITCN-BiGRU, and BITCN-Transformer models. The BITCN-MixedSSM model consistently outperformed other approaches, exhibiting superior accuracy across diverse temporal scales and power generation levels. This model’s efficacy is particularly evident in its ability to capture both peak and trough values with remarkable precision, suggesting an enhanced capacity to model both short-term fluctuations and long-term dependencies in the time series data. The CNN-LSTM and BITCN-LSTM models displayed stable prediction curves throughout the time series, albeit occasionally missing abrupt changes. In contrast, while the BITCN-MixedSSM model more closely approximated actual values overall, it exhibited minor fluctuations at certain time points. Notably, all models encountered challenges in predicting extreme anomalies, such as unusually high peaks, though the BITCN-MixedSSM demonstrated superior recovery speed, indicating robust resilience to disturbances. The model’s consistent performance across various temporal resolutions, from hourly to daily scales, underscores its adaptability to different time scales. These findings suggest that the BITCN-MixedSSM model’s architecture, which integrates the temporal convolutional properties of BITCN with the long and short-term memory capabilities of MixedSSM, confers a significant advantage in solar power prediction tasks. However, the observed challenges in extreme scenarios for all models highlight the potential benefit of multi-model ensemble approaches or the incorporation of additional external factors to enhance prediction robustness in practical applications further.

### 4.7. Data Fusion and PV Power Prediction

#### 4.7.1. K-Means++ Data Fusion

The accuracy of probabilistic forecasting for PV power generation is influenced by three critical factors: the precision of weather forecasts at the plant location, the availability of real-time power generation data from the plant, and the potential improvement of meteorological variable measurements at the site. Additionally, the effectiveness of the forecasting algorithms is critical. This study adheres to these three data criteria, and one of its objectives is to analyze the relative importance of these factors in predicting the quality of the forecast.

**Figure 10:**
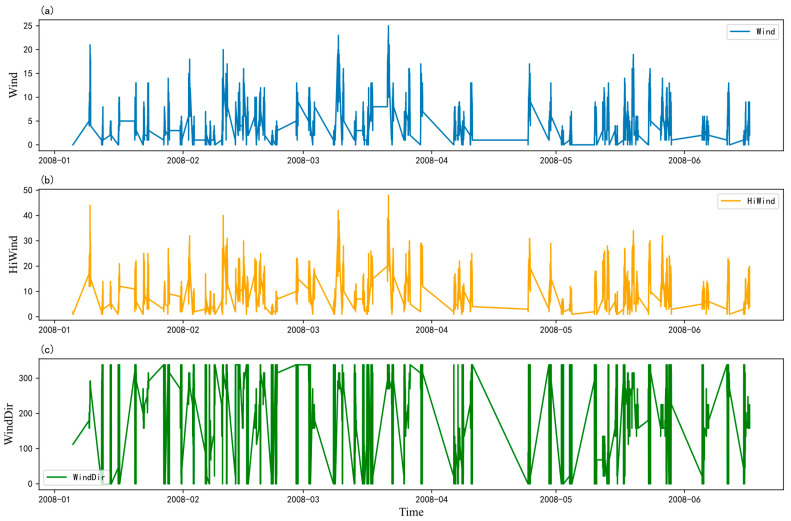
Graphical representation of the raw dataset for PV power prediction with (**a**) wind speed data, (**b**) maximum wind speed data, and (**c**) wind direction data.

In this paper, data from the original PV power prediction dataset’s full duration is analyzed, focusing on three key variables: wind speed, maximum wind speed, and wind direction. The raw, unfused data is presented in Figure 10. As demonstrated in Figure 10a, the wind speed data, and Figure 10b, the maximum wind speed data, both exhibit notable consistency, displaying periodic fluctuations with cycles lasting between 50 and 70 min. From 10 April 2008 to 2 May 2008, wind speed maintained a relatively steady rate, in alignment with the regular variations in wind direction during that period. Figure 10c demonstrates that wind direction also follows a periodic pattern, with more significant variation observed between 10 January 2008 and 28 February 2008. During the period from 10 April 2008 to 2 May 2008, wind direction changes more slowly, showing a linear and orderly periodic trend.

**Figure 11:**
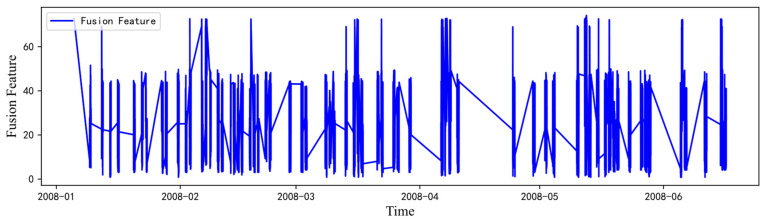
Graphical representation of the data after fusion feature processing of the raw dataset for PV power generation prediction.

The K-Means++ algorithm performs clustering by calculating the Euclidean distance between data points and the initial centroids. It is known for its fast computation and high efficiency. Unlike the traditional K-Means algorithm, K-Means++ improves the selection of cluster centroids by avoiding random initialization, resulting in better clustering outcomes. The fusion of wind speed, maximum wind speed, and wind direction data is illustrated in Figure 11. This data fusion somewhat enhances the overall accuracy and reveals more distinct periodic patterns. This study uses the fused time series of wind speed, maximum wind speed, and wind direction as a separate feature sequence, thus constructing new input time series samples. Additionally, it is observed that the resulting fused feature graph closely aligns with the wind direction data, indicating that the clustering process tends to favor the wind direction sequence.

#### 4.7.2. Fusing Different Input Features to Predict

In this section, we validate the use of the K-Means++ algorithm to process combinations of varying numbers of input features, aiming to develop a fused feature input model. The original PV power prediction dataset includes nine input features and one target feature. Based on Spearman coefficient analysis, humidity, and precipitation are fused using the K-Means++ algorithm, resulting in eight input features: temperature, dew point, wind speed, maximum wind speed, wind direction, atmospheric pressure, surface evaporation, and the fused feature. Temperature, wind speed, and maximum wind speed, which carry the highest weight according to the Spearman coefficient, are also fused using K-Means++, resulting in seven input features (7A): humidity, dew point, wind direction, precipitation, atmospheric pressure, surface evaporation, and the fused feature. Another set of seven input features (7B) is created by fusing wind speed, maximum wind speed, and wind direction, leaving temperature, humidity, dew point, precipitation, atmospheric pressure, surface evaporation, and the fused feature. Spearman coefficients are divided into positive and negative correlations, and input features are fused accordingly, resulting in two input features: one for positively correlated and one for negatively correlated variables.

The experimental results, as shown in Table 4, indicate that the 7A input feature set achieved the best performance, with an MAE of 0.065, RMSE of 0.087, and an *R*^2^ of 0.869, improving the *R*^2^ by 8.49% compared to the original nine input features. The 7B feature set showed the second-best performance, improving the *R*^2^ by 6.62%. Notably, humidity and precipitation, which exhibit a negative correlation based on the Spearman coefficient, show enhanced performance when fused, improving the *R*^2^ by 1.37% over the original nine features. This suggests that data fusion mitigates the negative correlation between humidity and precipitation, providing performance gains with other input variables. However, directly fusing positively and negatively correlated features resulted in significant performance degradation, with the *R*^2^ decreasing by 29.71%, indicating that such fusion is detrimental and disrupts the relationships between the original input features.

### 4.8. Robustness Verification

Due to the randomness, volatility, and intermittency of renewable energy generation, the resilience of prediction models in the presence of erroneous or noisy data becomes crucial [40]. Data from February 2008 to March 2008 is selected as the target dataset. Based on Spearman correlation analysis, Gaussian noise is added to the ET, Wind, HiWind, WindDir, and Barom data columns to simulate anomalous data, as these variables are positively correlated with photovoltaic power generation. As shown in Table 5, when noise is added only to the ET variable, the model demonstrates stable performance, consistent with its performance without noise. As noise is introduced into the Wind variable, model performance slightly degrades (RMSE increases to 0.110, *R*^2^ drops to 0.803). Further noise inclusion in the HiWind and WindDir variables results in a continued minor decline in performance. Notably, even after adding noise to these additional features, the model maintains relatively stable performance (RMSE at 0.122, *R*^2^ at 0.782), highlighting its robustness to noise. Interestingly, when Barom alone is used as the noisy input, the model performs better than when Wind is used. This suggests that noisy pressure data may provide more reliable or relevant information compared to noisy wind speed data.

## 5. Conclusions

This study proposes a BiTCN-MixedSSM model that integrates meteorological parameters to predict PV power generation and conducts a detailed performance analysis. The proposed model enhances the bidirectional temporal convolutional network (BiTCN) using dynamic convolution and employs BiLSTM to capture longer-range dependencies extracted by BiTCN. Additionally, a novel MixedSSM is designed by combining the state space model (SSM), multilayer perceptron (MLP), and multi-head self-attention (MHSA) modules to process various feature patterns in time series data. This approach enables flexible nonlinear mapping and robust contextual modeling. The effectiveness of the model for PV power forecasting is demonstrated through experimental validation. Results show that the model outperforms others in prediction accuracy, with an RMSE of 0.112 and an *R*^2^ of 80.1%, highlighting its potential for real-world applications. Spearman correlation was used to extract relevant features, and the model’s performance was compared across different models, identifying the optimal combination of input features. Additionally, the performance differences between related models were explored by training a BiTCN-MixedSSM model with full input features. Comparative analysis of univariate and multivariate inputs further confirmed the effectiveness of the hybrid CNN-LSTM model. In the experiments, the K-Means++ algorithm was employed for data fusion, and the model was trained using the fused features. Comparing the fused input features with those that were either unfused or improperly fused, it was confirmed that the newly derived features significantly impacted PV power prediction accuracy. The results demonstrate that fusion techniques notably enhance prediction performance, especially when handling negatively correlated features like humidity and precipitation.

To comprehensively assess the model’s performance, BiTCN-MixedSSM consistently demonstrated high prediction accuracy across all input feature combinations. Notably, the model achieved the highest *R*^2^ of 0.869 after applying data fusion, further validating its superiority in PV power forecasting. The study also examined the influence of different input feature combinations on model performance, finding that the fusion of positively and negatively correlated features significantly affects the model’s accuracy. Directly merging positively and negatively correlated features can disrupt the intrinsic relationships within the data, leading to a reduction in predictive accuracy. Therefore, careful handling of feature correlations is necessary during data fusion to optimize model performance. In conclusion, the BiTCN-MixedSSM model integrates various advanced deep learning techniques and has been empirically validated for PV power prediction. The study highlights the crucial role of data fusion in enhancing model accuracy and offers valuable insights into the impact of different input feature combinations on performance. This research provides a novel approach to PV power forecasting and contributes to advancing clean energy development.

## Figures and Tables

**Table 1:** Performance of multiple modeled PV generation forecasts on a test dataset.

Metrics	LightGBM	XGBoost	Ours	LSTM	1DCNN	BPNN	DT	RF
MAE	0.261	0.347	0.011	0.234	0.233	0.333	0.315	0.258
RMSE	0.174	0.269	0.012	0.125	0.155	0.147	0.326	0.345
*R* ^2^	0.675	0.689	0.891	0.812	0.733	0.709	0.605	0.625

**Table 2:** Experimental results of BiTCN-MixedSSM for feature selection for PV power prediction.

Input Feature	MAE	RMSE	*R* ^2^
experimental 1	0.077	0.112	0.801
experimental 2	0.074	0.101	0.811
experimental 3	0.070	0.081	0.812
experimental 4	0.071	0.091	0.808
experimental 5	0.045	0.065	0.826
experimental 6	0.041	0.066	0.824
experimental 7	0.052	0.072	0.818
experimental 8	0.011	0.012	0.891

**Table 3:** Experimental results of BiTCN-MixedSSM and its related models for PV power generation prediction.

Model	MAE	RMSE	*R* ^2^
RNN	0.233	0.402	0.618
LSTM	0.197	0.304	0.705
CNN-LSTM	0.185	0.285	0.711
ARIMA	0.212	0.395	0.635
GARCH	0.201	0.327	0.687
BiTCN-BiGRU	0.121	0.178	0.772
BiTCN-Transformer	0.098	0.147	0.792
BiTCN-SVM	0.159	0.254	0.759
BiTCN-LSTM	0.137	0.194	0.765
BiTCN-MixedSSM	0.077	0.112	0.801

**Table 4:** Experimental results on the performance of data fusion using K-Means++ algorithm for PV power generation prediction.

Input Feature Number	BiTCN-MixedSSM		
	MAE	RMSE	*R* ^2^
9	0.077	0.112	0.801
8	0.072	0.101	0.812
7A	0.065	0.087	0.869
7B	0.071	0.092	0.854
2	0.389	0.412	0.563

**Table 5:** Proposed BiTCN-MixedSSM validates robustness by noisy dataset.

ET	Wind	HiWind	WindDir	Barom	RMSE	*R* ^2^
✓					0.101	0.811
	✓				0.110	0.803
	✓	✓			0.119	0.795
	✓	✓	✓		0.122	0.782
				✓	0.111	0.799
✓				✓	0.120	0.789

## Data Availability

The data are available from the authors upon reasonable request.

## Abbreviations

The following abbreviations are used in this manuscript:

PV	photovoltaic
BiTCN	bidirectional temporal convolutional networks
BiLSTM	long short-term memory networks
DC	dynamic convolution
SSM	state space model
MLP	multilayer perceptron
MAE	mean absolute error
RMSE	root mean squared error
ANNs	artificial neural networks
ViT	vision transformer
MHSA	multi-head self-attention
CSM	cross-scanning module
ML	machine learning
